# Experimental evidence challenges the presumed defensive function of a “slow toxin” in cycads

**DOI:** 10.1038/s41598-022-09298-3

**Published:** 2022-04-09

**Authors:** Melissa R. L. Whitaker, Florence Gilliéron, Christina Skirgaila, Mark C. Mescher, Consuelo M. De Moraes

**Affiliations:** Department of Environmental Systems Science, ETH Zürich, 8092 Zurich, Switzerland

**Keywords:** Biochemistry, Ecology, Plant sciences, Ecology

## Abstract

$\beta$-methylamino-L-alanine (BMAA) is a neurotoxic non-protein amino acid found in the tissues of cycad plants. The demonstrated toxicity of BMAA to diverse organisms, including humans, is widely assumed to imply a defensive function of BMAA against herbivores; however, this hypothesis has not previously been tested in an ecologically relevant system. We investigated the effects of dietary BMAA, across a range of dosages matching and exceeding levels typically present in cycad leaves, on the feeding preferences and performance of a generalist lepidopteran herbivore (*Spodoptera littoralis*).We observed no effects of dietary BMAA on the survival or development of *S. littoralis* larvae, nor any larval preference between BMAA-laced and control diets. These findings suggest that BMAA in cycad tissues does not deter feeding by insect herbivores, raising questions about other potential physiological or ecological functions of this compound.

## Introduction

The non-proteinogenic amino acid $\beta$-methylamino-L-alanine (BMAA) has received considerable attention due to its implication in several human neurodegenerative disorders^1^. BMAA is naturally produced by cyanobacteria living in diverse environments^2–4^ and can bioaccumulate within aquatic and terrestrial food webs^5^, such that humans can be exposed to BMAA through environmental and dietary routes^6,7^. In addition, BMAA, possibly originating from cyanobacterial symbionts, appears to be universally present in the tissues of cycad plants^8^, a traditional food source for many human populations living in tropical regions. While the functional significance of BMAA in cycad tissues has yet to be definitively established, it is widely assumed that BMAA functions as a defensive compound deterring insect herbivory. However, this hypothesis has not previously been tested in an ecologically relevant system.

In general, the biological and ecological functions of BMAA for cycads remain uncertain: although some metabolic and signaling functions have been proposed, the most prevalent hypothesis for an adaptive function of BMAA in cycads is that it confers protection against herbivory, consistent with its demonstrated toxicity to diverse organisms including non-human primates and other mammals^9–11^, crustaceans^3^, fish^12^, and microbes^13^. Indeed, numerous papers implicitly or explicitly refer to BMAA as an anti-herbivore compound^14–22^. Nevertheless, empirical evidence supporting a defensive function of BMAA in cycads is lacking, particularly with respect to what are likely to be the most relevant herbivores of cycads: grazing insects.

A few studies have examined the effects of BMAA ingestion in other insect groups. For example, *Drosophila melanogaster* fed on BMAA exhibit shorter lifespans, reduced neurological function, and severe motor impairment as adults^23–26^. Similarly, adult honeybees (*Apis mellifera*) experience higher mortality and decreased neurological functions when fed BMAA^27^. While these results are consistent with a defensive function of BMAA, neither fruit flies nor honeybees are natural herbivores of cycads, and the dietary BMAA doses employed in these studies exceed quantities that naturally occur in most cycad tissues. A further issue with interpreting BMAA’s toxicity as evidence for an anti-herbivore function is that the onset of negative effects is typically significantly delayed following exposure. BMAA has been characterized as a “slow toxin”^28^, and in humans, years or decades can pass between exposure to BMAA and disease onset. Such long delays in the manifestation of toxic effects, which have also been observed in other organisms, including *Drosophila*^24^, would seemingly undermine BMAA’s efficacy as a short-term feeding deterrent.

Thus, a more conclusive evaluation of the putative defensive function of BMAA requires not only examining its effects within an ecologically relevant system but also evaluating the implications of BMAA ingestion for short-term herbivore feeding preferences and performance. The present study aims to achieve this by evaluating whether ecologically relevant doses of BMAA (comparable to those found in cycad leaves) are sufficient to deter herbivory by a generalist lepidopteran herbivore, *Spodoptera littoralis*, using classic preference-performance bioassays. To our knowledge, this represents the first experimental test of BMAA’s defensive potential.

## Methods

### Study organisms

We used *Spodoptera littoralis*, a generalist lepidopteran herbivore and established model species, to test the effects of dietary BMAA on herbivore survival, development, and feeding preferences. This species was selected due to its broadly polyphagous diet: *S. littoralis* has been recorded feeding on more than 100 plant species from 49 different families^29^. Although there are no records of *S. littoralis* feeding on cycads, this species is broadly similar to other generalist herbivores in its sensitivity to plant defenses and is commonly used in preference-performance bioassays^30^. *Spodoptera littoralis* eggs were obtained from Syngenta International AG in Basel, Switzerland, and were incubated at 28 °C for 48 h until hatching, after which the larvae were reared at 27 °C, 60% RH and a 16:8 L:D cycle. This combination of hatching/rearing temperatures provides better control over experimental timing.

### Developmental assay

Upon hatching, larvae were moved to plastic rearing cups containing a small cube of artificial diet (Frontier Insect Diets, product F9772) augmented with BMAA (Sigma Aldrich, CAS Number 16012-55-8) at varying concentrations within the range of reported BMAA levels in cycad leaves^8,31,32^: 10 $\upmu$g/g, 50 $\upmu$g/g, and 250 $\upmu$g/g, along with a BMAA-free control. Thirty-five larvae were assigned to each treatment, reared individually, and fed *ad libitum*. Food was weighed to allow estimation of the total quantity ingested by each larva and was refreshed daily as needed. Dates of moulting (indicated by the presence of a shed exoskeleton and head capsule) were recorded. Prepupae were transferred individually into fresh rearing cups containing only filter paper. Upon pupation, pupae were weighed and sexed (under a dissecting microscope), and dates of pupation and adult emergence were recorded.

All statistical analyses were conducted in R version 3.5.1^33^. Survival data were analyzed with the survival^34^ and survminer^35^ packages using a log rank test, and time-to-pupation data were analyzed using a cox proportional hazards model after confirming the data met the proportional hazards assumption. Pupal weight was compared via one-way ANOVA after confirming normality and accounting for treatment effects, sex effects, and the treatment*sex interaction. Data for total food consumption were not normally distributed and were therefore compared via a Kruskal–Wallis test. To test whether BMAA induced sex-specific differences in mortality, we compared sex ratios for the two treatments that experienced larval mortality using a two-proportions z-test.

### Choice assay

*Spodoptera littoralis* larvae were reared from eggs in communal rearing containers on one of two artificial diet treatments using the same artificial diet as above: diet spiked with 250 $\upmu$g/g BMAA and a BMAA-free control diet. Twenty-seven larva were reared of BMAA-spiked diet, and 32 larvae were reared on control diet. After feeding for 10 days, individual larvae were placed in petri dishes with small cubes ($\sim$0.5 g) of each diet placed on opposite sides of the dish. Dishes were arranged in an arena and filmed from above for 24 h using Raspberry Pi model 3B+ computers fitted with IR-CUT infrared cameras filming in night mode at a framerate of 25 frames per second.

**Figure 1:**
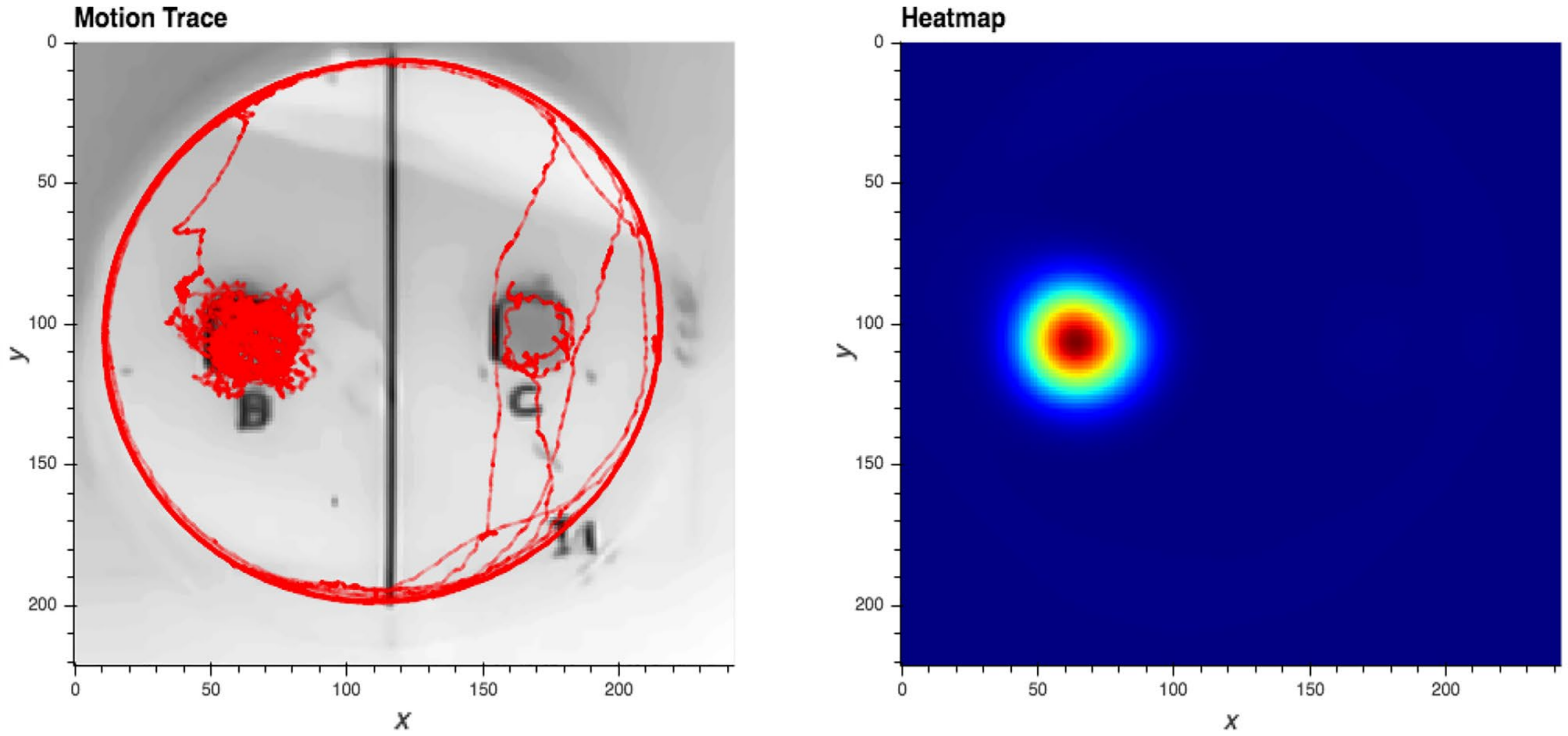
ezTrack output: a motion trace of a single larva (left), showing that the larva encountered both diet cubes but spent significantly more time on one, and a heatmap (right) based on aggregated location data from the same trial.

Five larvae spent less than 1 h in contact with either diet cube during the experiment and were therefore excluded from data analyses (two control-reared larvae and three BMAA-reared larvae). Video recordings for the remaining trials were analyzed using the python-based software package ezTrack^36^ executed within a Jupyter Notebook^37^. We constructed a reference frame for each trial by averaging 200 frames selected randomly by the software or, in cases where ezTrack could not calculate a reference frame because a larva occupied the same location for more than 50% of the video, 200 frames from a manually selected video segment. In cases where larvae were mostly sedentary, videos were analyzed manually. The software produced a heatmap and tracking map (Fig. 1) for each trial, along with frame-by-frame tracking of each larva’s location within the petri dish. An example analysis notebook is provided in the Supplemental Information.

For each larva, a preference index was calculated as the time spent on BMAA-spiked diet divided by the time spent on either diet, with a preference index greater than 0.5 indicating a preference for BMAA-spiked diet and an index less than 0.5 indicating a preference for the control diet. Preference indices were compared using an exact binomial test. The effects of larval treatment and first dietary choice on dietary preference were analyzed using a generalized linear model with a quasi-binomial distribution.

### Bioassays with higher BMAA dosage

To rule out the possibility that our highest dosage (250 $\upmu$g/g) was too low to elicit any effects, we repeated the above experiments with a second batch of larvae reared on a higher BMAA concentration of 2000 $\upmu$g/g along with a second control (no BMAA). This dosage corresponds to typical BMAA levels found in cycad seeds, but is much higher than levels reported from the leaves of most cycads^31^.

## Results

### Developmental assay

**Figure 2:**
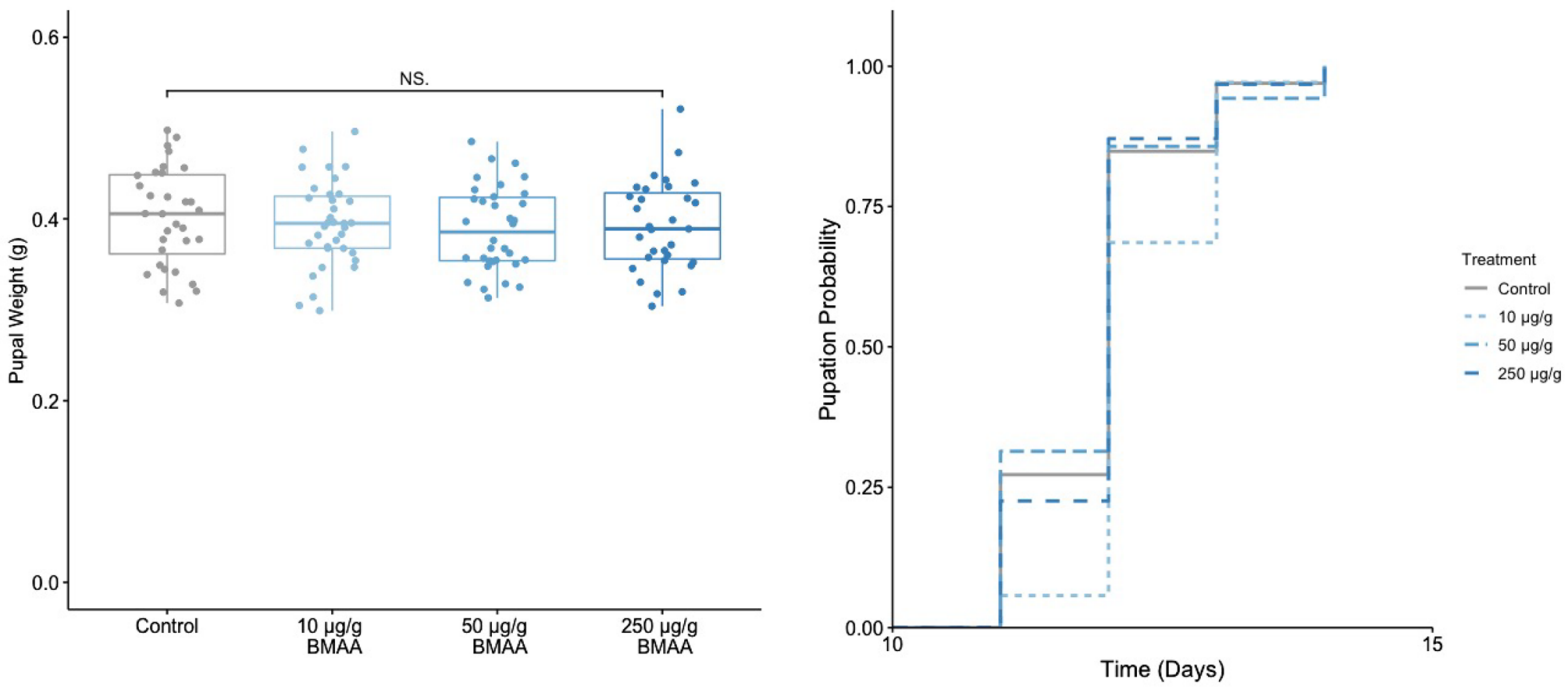
Pupal weight (left) and time to pupation (right) did not differ among *Spodoptera littoralis* reared on different BMAA treatments.

Average larval food consumption was 3.836 g, corresponding to lifetime BMAA intake of $\sim$38 $\upmu$g, $\sim$192 $\upmu$g, and $\sim$959 $\upmu$g for the corresponding BMAA treatments. Total food consumption did not differ between treatments (H = 3.28, p = 0.35). Larval survival was high overall, with 97% of larvae reaching the adult stage, and exhibited no significant differences among treatments (p = 0.22). Similarly, BMAA consumption did not affect sex ratios (*X*^2^(1, N = 43) = 0.39, p = 0.53), time to pupation (Fig. 2; p = 0.89, Supplemental Table 1), or pupal weight (Fig. 2; F(3,124) = 0.61, p = 0.61), though males were significantly smaller than females across all treatments (F(1,124) = 53.21, p < 0.001).

### Preference assay

**Figure 3:**
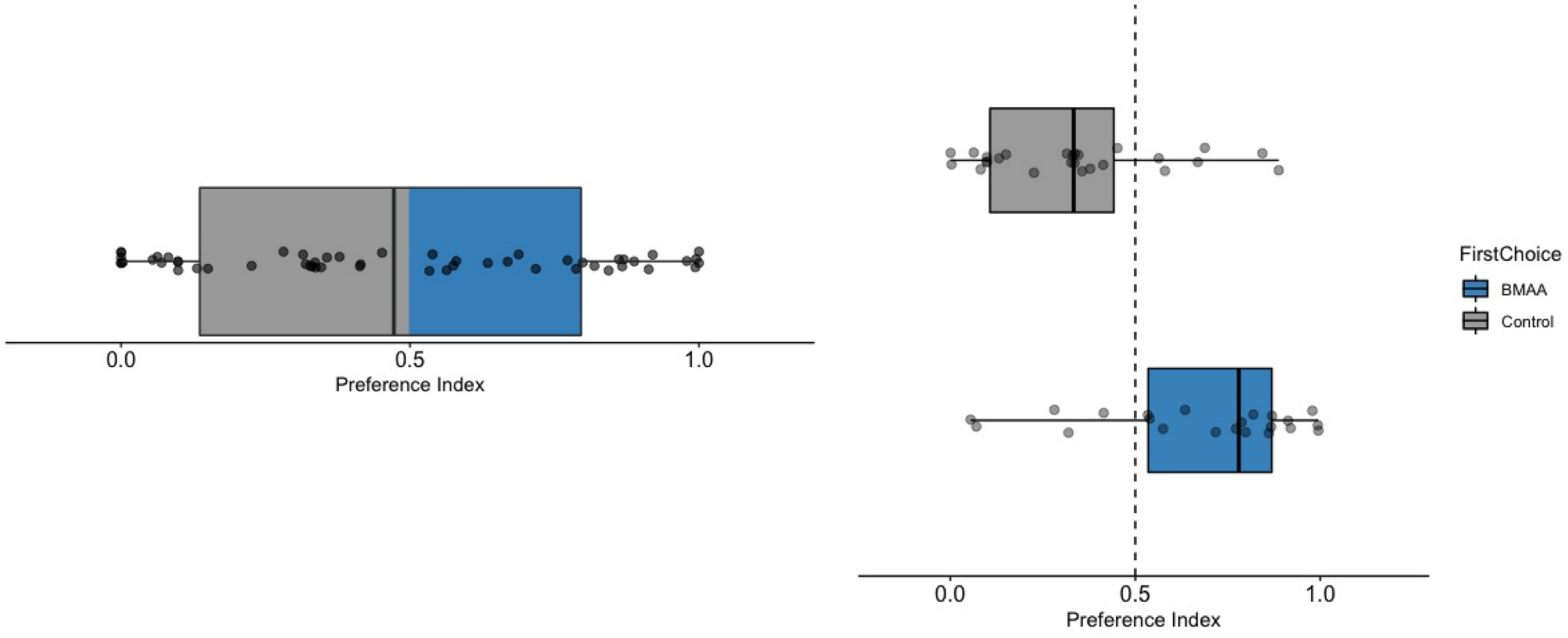
Larval preference indices indicate preference for BMAA-spiked vs. control diets. Although there was no significant preference for control vs. BMAA-spiked diet overall (left), larvae strongly preferred whichever cube of diet they encountered first (right), even if they switched between diets over the course of the trial.

Over 100 million video frames were analyzed using ezTrack. Most larvae spent more than 50% of the trial period in direct contact with a diet cube, but larvae often switched between cubes and also explored the dishes. Larvae did not exhibit a significant preference for BMAA-spiked or control diet (Fig. 3, p = 0.5), and larval rearing treatment did not affect larval preferences (t(53) = $-0.69$, p = 0.49) nor the total time larvae spent feeding (F(1,52) = 1.61, p = 0.21). Larvae tended to prefer the diet cube they encountered first during the trial period, spending significantly more time on their first choice even if they subsequently fed on the other cube as well (t(47) = $-4.15$, p < 0.001; Fig. 3), but overall, larvae did not spend significantly more time feeding on either diet (*X*^2^(1, N = 54) = 0.67, p = 0.41).

### Bioassays with higher BMAA dosage

We observed significant differences in development time and pupal weight between batches of larvae, with the second batch exhibiting slightly lower survival rates, taking approximately 1 week longer to pupate, and pupating at roughly half the size of larvae from the first batch. Though we cannot explain these differences, such “batch effects” are common for insects reared in captivity. Importantly, we detected no significant effects of dietary BMAA on larval preference or performance within either batch (Supplemental Information).

## Discussion

Our experiments revealed no preference of *Spodoptera littoralis* larvae for artificial diet with or without BMAA. Furthermore, BMAA ingestion had no effect on larval development or survival, even at concentrations much higher than those typically present in cycad leaves. The findings indicate that dietary exposure to BMAA is insufficient to deter feeding by grazing insects and thus challenge the widespread view that BMAA functions as a defensive compound in the context of cycad-insect interactions.

Our results do not exclude a possible role for BMAA in plant defense against other cycad antagonists, including vertebrate herbivores, microbial pathogens, or other insect herbivores. Certain physiological attributes of Lepidopteran larvae, including relatively short food retention times and highly alkaline guts with continuously replaced lining^38^, could plausibly mitigate toxic effects of exposure to BMAA, in which case insects with different gut physiology may be more vulnerable. Future feeding assays could be performed with herbivores from other insect orders, especially Coleoptera, to test whether BMAA exhibits herbivore-specific defensive effects. Cycad leaves also contain numerous other secondary metabolites that were absent from the diets employed in our experiments, and we cannot exclude potential synergistic effects of BMAA in combination with other plant compounds. While most previous investigations of cycad defensive chemistry have focused on BMAA, along with methylazoxymethanol glycosides, future work would do well to elucidate the distribution and defensive potential of the many other compounds found in cycad tissues.

These caveats notwithstanding, we feel the weight of available evidence bears strongly against an anti-herbivore function of BMAA, based both on the absence of short-term effects in the current study and on the extensive latency between exposure and the onset of toxic effects observed in other animals^28^. In mammals, for example, BMAA exposure at a young age can cause neurological symptoms that become apparent only much later in life^11,39^. Similarly, fruit fly larvae reared on BMAA-laced diet exhibited delayed locomotive impairment as adults, even though BMAA did not negatively affect larval survival^23^. Similar long-term effects of BMAA exposure on lepidopterans or other insect herbivores are not excluded by the current findings; however, given that most adult Lepidoptera reproduce within a few days of eclosion, symptoms arising in adults would likely manifest too late to prevent a new generation of larvae. Furthermore, for at least one cycadivorous lepidopteran species, a single larval generation can severely defoliate a large cycad^17,40^, suggesting that defenses would need to be fast-acting in order to be effective.

It has, however, been suggested that delayed plant defenses could be adaptive under certain conditions. Specifically, Backmann et al. (2019)^41^ proposed that plants facing strong intraspecific competition might optimize fitness by delaying the induction of chemical defenses until herbivores are large enough to disperse to neighboring plants. While there is currently no evidence that BMAA production is inducible in cycads, the possibility cannot be discounted. However, the current findings raise doubts about whether exposure even to high concentrations of BMAA would deter feeding or cause dispersal. Furthermore, many cycad species do not grow in dense cohorts^42^, and Backmann et al.’s results suggest that, in the absence of strong intraspecific competition, chemical defenses should be deployed as rapidly as possible. In the case of lepidopteran herbivores, plant defenses are likely to be most effective at reducing damage when acting within the 1–2 weeks required for most caterpillars to reach late instars, when they are most damaging to plants.

Failure to find evidence supporting anti-herbivore effects, at least against generalist insects, has implications for future investigations into the chemical ecology of cycad-insect and cycad-cyanobacteria interactions. Cycad defensive toxins have been proposed to be important drivers of trophic specialization among cycadivorous insects^19,21,43^, and our results suggest that BMAA is unlikely to be a key compound mediating cycads’ interactions with herbivores over ecological or evolutionary timescales. Based on abundant evidence that microbial symbionts can enhance anti-herbivore defenses in other plants^44^, it is also plausible that some cyanobacteria-derived metabolites—including BMAA—could have cascading effects on higher trophic levels. However, the current findings suggest that if BMAA plays an important role in the mutualistic relationship between cyanobacteria and cycads, it does so via mechanisms other than the enhancement of plant defenses.

The current findings also highlight the need to consider and test alternative hypotheses regarding the functional significance of BMAA for cycads. While a few alternative functions related to metabolism and signaling have been proposed^18^, we are not aware of any experimental work investigating potential physiological or ecological functions. Fortunately, experimental tests such as the preference-performance assays utilized in the present study are inexpensive and straightforward, though they do require chemical standards of the compound(s) of interest and some a priori knowledge regarding ecologically relevant doses and plausible herbivores. Our experiments leveraged model organisms, Raspberry Pi computers, and open-source software to create a reproducible experimental workflow available to researchers interested in conducting similar studies.

Future studies addressing the role of BMAA in cycads would do well to place greater emphasis on the identification and elucidation of non-defensive functions. Unfortunately, functional studies in cycads have proved challenging, in part because the life histories of these plants, which tend to be slow-growing, are not amenable to experimental manipulation, and because a lack of genomics resources for cycads precludes the use of most molecular tools. Elucidating BMAA’s functions in cyanobacteria may therefore provide a more useful starting point for guiding studies in cycads. Several ecophysiological functions of BMAA have been proposed for cyanobacteria (reviewed in^45^), some of which might also be relevant for regulating cycads’ responses to biotic and abiotic stressors such as competition, nitrogen limitation, or intense light conditions. As of this writing, however, BMAA’s presumably adaptive function—as well as the mechanisms of its production, transport, and storage—remain unsolved problems in cycad biology.

## Supplementary Information

Supplementary Information 1.

Supplementary Information 2.
