# Supplementary material for: Experimental evidence challenges the presumed defensive function of a “slow toxin” in cycads
Source: Sci Rep. 2022 Apr 9;12:6013. doi: 10.1038/s41598-022-09298-3 (PMC8994766; doi:10.1038/s41598-022-09298-3)
Supplement: Supplementary file 1 — Supplementary Information 1. [file 41598_2022_9298_MOESM1_ESM.pdf]

Preamble

This is a modified version of the LocationTracking_Individual.ipynb file from ezTrack. To use this, you must download and install ezTrack according to the instructions at https://github.com/denisecailab/ezTrack with all dependencies. Then move this file and the ModifiedLocationTracking_Functions.py script into your ezTrack directory to run.

In example given below, 24-hour videos had been split into 3 8-hour time segments and each video segment is analyzed separately. You must modify the code according to the number of video files (per behavioral trial) you are analyzing. The analysis will need to repeated for every behavioral trial.

Overview

1. Set up / load packages
2. Load videos
3. Crop video
4. Confirm reference frames
5. Define regions of interest
6. Confirm tracking in a few frames
7. Conduct tracking in all frames (computationally intensive)
8. Export location data (csv and heatmap)

0. Set up / Load Necessary Packages

In [ ]:
```
import os
import cv2
import numpy as np
import matplotlib.pyplot as plt
import pandas as pd
import ModifiedLocationTracking_Functions as lt
import holoviews as hv
from bokeh.io import export_png
from sinfo import sinfo
import more_itertools as mit
sinfo()
%load_ext autoreload
%autoreload 2
```

1. Load Video File(s)

In [ ]:
```
#This part is optional and should be adapted to your trial naming scheme
Pi='1'
Dish='A'
TrialID = (f'{Pi}.{Dish}')
```

In [ ]:
```
video_dict1 = {
    'fpath' : f"/Full/Path/To/Video/File/file.avi",
    'dpath' : f"/Full/Path/To/Video/File/",
    'file' : f"file.avi",
    'start' : 0,
    'end' : None,
    'dsmpl' : 1,
    'TrialID' : TrialID, #(optional)
    'part' : '_part1' #(optional)
}
video_dict2 = {
    'fpath' : f"/Full/Path/To/Video/File/file.avi",
    'dpath' : f"/Full/Path/To/Video/File/",
    'file' : f"file.avi",
    'start' : 0,
    'end' : None,
    'dsmpl' : 1,
    'TrialID' : TrialID, #(optional)
    'part' : '_part2' #(optional)
}

video_dict3 = {
    'fpath' : f"/Full/Path/To/Video/File/file.avi",
    'dpath' : f"/Full/Path/To/Video/File/",
    'file' : f"file.avi",
    'start' : 0,
    'end' : None,
    'dsmpl' : 1,
    'TrialID' : TrialID, #(optional)
    'part' : '_part3' #(optional)
}


tracking_params = {
    'loc_thresh' : 98, #this threshhold can be increased or decereased; see ezTrack documentation
    'use_window' : True,
    'window_size' : 100,
    'window_weight' : .9,
    'method' : 'abs' #see other options in ezTrack documentation
}

stretch = dict(width=1, height=1)
region_names = ['BMAA_food', 'Control_food', 'BMAA_side','Control_side'] #names of Regions of Interest
```

2. Crop Frame

In [ ]:
```
%%output size=100

image,crop,video_dict1 = lt.LoadAndCrop(video_dict1,stretch,cropmethod='Box')
image
```

Save the crop coordinates (optional)

In [ ]:
```
print(crop.data)
```

3. Create Reference Frames For Each Video Segment

Video 1

In [ ]:
```
%%output size=200

# There are two options for making reference frames from video files for which you did not pre-record a dedicat
# reference frame: the first is an automated approach in which eZTrack will randomly select a number of frames
# (given by 'num_frames') from the video and create an average of them. This works well for animals
# that move consistently throughout the video and do not spend more than 50% of the time in one location.

reference1, image = lt.Reference(video_dict1,stretch,crop=crop,num_frames=200,frames=None)

# The second option is to select frames manually by using the 'frames=np.arange()' option with three parameters
# the first value is the frame to start with, the second value is the frame to end with, and the third value is
# how often to select a frame within the range (which should be selected so that the total number of frames equ
# 'num_frames' from the previous option). This option works if, for example, an animal is active in the first t
# hours only so the reference frame should be calculated from that time window.

reference1, image = lt.Reference(video_dict1,stretch,crop=crop,frames=np.arange(0,200,1))

# whichever option you decide to use, just make sure you comment out the unused option

image
```

In [ ]:
```
#save reference frame (optional)
hv.save(image, TrialID + '_reference_part1.png')
```

Video 2

In [ ]:
```
%%output size=200

reference2, image = lt.Reference(video_dict2,stretch,crop=crop,num_frames=200,frames=None)

#OR

reference2, image = lt.Reference(video_dict2,stretch,crop=crop,frames=np.arange(0,360000,1800))


image
```

In [ ]:
```
#save reference frame (optional)
hv.save(image, TrialID + '_reference_part2.png')
```

Video 3

In [ ]:
```
%%output size=200

reference3, image = lt.Reference(video_dict3,stretch,crop=crop,num_frames=200,frames=None)

#OR

reference3, image = lt.Reference(video_dict3,stretch,crop=crop,frames=np.arange(360000,720000,1800))


image
```

In [ ]:
```
#save reference frame (optional)
hv.save(image, TrialID + '_reference_part3.png')
```

4. Define Regions of Interest

In [ ]:
```
%%output size=200

plot,poly_stream = lt.ROI_plot(reference1,region_names,stretch)
plot
```

In [ ]:
```
#save ROI data (optional)
print(poly_stream.data)
```

5. Confirm Location Tracking is Working as Expected

In [ ]:
```
%%output size = 100

examples = 5
images1=lt.LocationThresh_View(video_dict1,reference1,tracking_params,crop=crop,examples=examples,stretch=stret
images2=lt.LocationThresh_View(video_dict2,reference2,tracking_params,crop=crop,examples=examples,stretch=stret
images3=lt.LocationThresh_View(video_dict3,reference3,tracking_params,crop=crop,examples=examples,stretch=stret


ex1=images1.cols(2)
ex2=images2.cols(2)
ex3=images3.cols(2)

#save images (optional)
hv.save(ex1, TrialID +"_examples_part1.png")
hv.save(ex2, TrialID +"_examples_part2.png")
hv.save(ex3, TrialID +"_examples_part3.png")
```

In [ ]:
```
## Display images
```

In [ ]:
```
ex1
```

In [ ]:
```
ex2
```

In [ ]:
```
ex3
```

Note that image tracking in these examples is slightly less acurate than the full process (below), which incorporates information about an animal's previous location when calculating the location in the next frame. So don't be too worried by slight errors in these examples, just look out for consistent mistakes.

6. Full Location Tracking

In [ ]:
```
location1=lt.TrackLocation(video_dict1,tracking_params,reference1,crop=crop)
if region_names != None:
    location1 = lt.ROI_Location(reference1,location1,region_names,poly_stream)
if 'scale_dict' in locals():
    location1 = lt.ScaleDistance(scale_dict, dist, df=location1, column='Distance_px')
location1.to_csv(TrialID + video_dict1['part'] + '_LocationOutput.csv')

location2=lt.TrackLocation(video_dict2,tracking_params,reference2,crop=crop)
if region_names != None:
    location2 = lt.ROI_Location(reference2,location2,region_names,poly_stream)
if 'scale_dict' in locals():
    location2 = lt.ScaleDistance(scale_dict, dist, df=location2, column='Distance_px')
location2.to_csv(TrialID + video_dict2['part'] + '_LocationOutput.csv')

location3=lt.TrackLocation(video_dict3,tracking_params,reference3,crop=crop)
if region_names != None:
    location3 = lt.ROI_Location(reference3,location3,region_names,poly_stream)
if 'scale_dict' in locals():
    location3 = lt.ScaleDistance(scale_dict, dist, df=location3, column='Distance_px')
location3.to_csv(TrialID + video_dict3['part'] + '_LocationOutput.csv')

#examine the contents of the first file
location1.head()
```

7. Summarize Data

In [ ]:

When analyzing millions of video frames, there's a certain level of noise in the data and eztrack may erroneously identify some frames in which an animal is counted in multiple ROIs ("double counted" below) or is not counted in any ROI ("empty frames"). If double counted and empty frames are common, you should modify the tracking paramaters and try again. If they are uncommon they can be ignored, excluded, or smoothed in downstream data cleaning steps. Because the current workflow analyzed trials in three 8-hour segments, the following code includes steps to combine numbers from each segment, but this should be adjusted to reflect your own video files.

In [ ]:
```
tmpdf = {'TrialID':  [TrialID], 'RawTotalFrames': [len(location1)], 'BMAA_food': [location1.BMAA_food.sum()],'C
Adjusted=pd.DataFrame(tmpdf)

Adjusted['DoubleCounted_part1'] = ((location1['Control_side']==True) & (location1['BMAA_side']==True)).sum()
Adjusted['DoubleCounted_part2'] = ((location2['Control_side']==True) & (location2['BMAA_side']==True)).sum()
Adjusted['DoubleCounted_part3'] = ((location3['Control_side']==True) & (location3['BMAA_side']==True)).sum()
Adjusted['EmptyFrames_part1'] = ((location1['Control_side']==False) & (location1['BMAA_side']==False)).sum()
Adjusted['EmptyFrames_part2'] = ((location2['Control_side']==False) & (location2['BMAA_side']==False)).sum()
Adjusted['EmptyFrames_part3'] = ((location3['Control_side']==False) & (location3['BMAA_side']==False)).sum()
Adjusted['Problems'] = ((location1['Control_food']==True) & (location1['BMAA_food']==True)).sum()+((location2['
Adjusted['DoubleCounted_Total'] = Adjusted['DoubleCounted_part1'] + Adjusted['DoubleCounted_part2'] + Adjusted[
Adjusted['EmptyFrames_Total'] = Adjusted['EmptyFrames_part1'] + Adjusted['EmptyFrames_part2'] + Adjusted['Empty
Adjusted.to_csv(TrialID + '_SummarizedLocationData.csv') #this will create a single file for this behavioral tr
Adjusted.to_csv('MasterFile.csv', mode='a', index=False, header=False) #this will append to a master file conta
Adjusted.head()
```

8. Display Location

In [ ]:
```
%%output size=200

#Plot Distance Across Session
w, h = 600,600 #specify width and height of plot
dist_plot1 = hv.Curve((location1['Frame'],location1['Distance_px']),'Frame','Pixel Distance').opts(
    height=h,width=w,color='red',title="Distance Across Session",toolbar="below")
dist_plot2 = hv.Curve((location2['Frame'],location2['Distance_px']),'Frame','Pixel Distance').opts(
    height=h,width=w,color='red',title="Distance Across Session",toolbar="below")
dist_plot3 = hv.Curve((location3['Frame'],location3['Distance_px']),'Frame','Pixel Distance').opts(
    height=h,width=w,color='red',title="Distance Across Session",toolbar="below")

#Plot Trace of Animal Across Session and Generate Heatmap
tracks1 = lt.showtrace(reference1,location1,color="red",alpha=.05,size=2,stretch=stretch)
heatmap1 = lt.Heatmap(reference1, location1, sigma=None,stretch=stretch)
tracks2 = lt.showtrace(reference2,location2,color="red",alpha=.05,size=2,stretch=stretch)
heatmap2 = lt.Heatmap(reference2, location2, sigma=None,stretch=stretch)
tracks3 = lt.showtrace(reference3,location3,color="red",alpha=.05,size=2,stretch=stretch)
heatmap3 = lt.Heatmap(reference3, location3, sigma=None,stretch=stretch)

#Save files (optional)
hv.save(tracks1, TrialID + video_dict1['part'] + "_tracks.png")
hv.save(heatmap1, TrialID + video_dict1['part'] + "_heatmap.png")
hv.save(tracks2, TrialID + video_dict2['part'] + "_tracks.png")
hv.save(heatmap2, TrialID + video_dict2['part'] + "_heatmap.png")
hv.save(tracks3, TrialID + video_dict3['part'] + "_tracks.png")
hv.save(heatmap3, TrialID + video_dict3['part'] + "_heatmap.png")
```

9. Data Cleaning

In [ ]:
```
part1 = pd.read_csv(f'{wd}/{TrialID}_part1_LocationOutput.csv',index_col=0)
part2 = pd.read_csv(f'{wd}/{TrialID}_part2_LocationOutput.csv',index_col=0)
part3 = pd.read_csv(f'{wd}/{TrialID}_part3_LocationOutput.csv', index_col=0)

part1.head()
```

Make new data frame with average ROI locations (TRUE/FALSE) in *n* rowchunks, where *n* is the framerate of your video recordings

In [ ]:
```
part1["BMAA_food"] = 1 * part1["BMAA_food"]
part1["Control_food"] = 1 * part1["Control_food"]

part2["BMAA_food"] = 1 * part2["BMAA_food"]
part2["Control_food"] = 1 * part2["Control_food"]

part3["BMAA_food"] = 1 * part3["BMAA_food"]
part3["Control_food"] = 1 * part3["Control_food"]

part1=part1.groupby(np.arange(len(part1))//25).mean().round().astype(int)
part2=part2.groupby(np.arange(len(part2))//25).mean().round().astype(int)
part3=part3.groupby(np.arange(len(part3))//25).mean().round().astype(int)

part1.head()
```

Check quality

In [ ]:
```
part1['DoubleCountedFood'] = np.where((part1['BMAA_food']==1) & (part1['Control_food']==1), 'X', '')
part1['DoubleCountedSide'] = np.where((part1['BMAA_side']==1) & (part1['Control_side']==1), 'X', '')
part1['EmptyFrame'] = np.where((part1['BMAA_side']==0) & (part1['Control_side']==0), 'X', '')
print(f"Double Counted sides: {(part1.DoubleCountedSide == 'X').sum()}")
print(f"Double Counted food: {(part1.DoubleCountedFood == 'X').sum()}")
print(f"Empty seconds: {(part1.EmptyFrame == 'X').sum()}")
print(f"Total seconds: {len(part1)}")
firstBMAA = part1[(part1.BMAA_food == 1)].index[0]#these two lines could change depending on how many video seg
firstControl = part1[(part1.Control_food == 1)].index[0]#they may need to be moved to subsequent video segments
part1.head()
```

In [ ]:
```
part2['DoubleCountedFood'] = np.where((part2['BMAA_food']==1) & (part2['Control_food']==1), 'X', '')
part2['DoubleCountedSide'] = np.where((part2['BMAA_side']==1) & (part2['Control_side']==1), 'X', '')
part2['EmptyFrame'] = np.where((part2['BMAA_side']==0) & (part2['Control_side']==0), 'X', '')
print(f"Double Counted sides: {(part2.DoubleCountedSide == 'X').sum()}")
print(f"Double Counted food: {(part2.DoubleCountedFood == 'X').sum()}")
print(f"Empty Frames: {(part2.EmptyFrame == 'X').sum()}")
print(f"Total frames: {len(part2)}")
part2.head()
```

In [ ]:
```
part3['DoubleCountedFood'] = np.where((part3['BMAA_food']==1) & (part3['Control_food']==1), 'X', '')
part3['DoubleCountedSide'] = np.where((part3['BMAA_side']==1) & (part3['Control_side']==1), 'X', '')
part3['EmptyFrame'] = np.where((part3['BMAA_side']==0) & (part3['Control_side']==0), 'X', '')
print(f"Double Counted sides: {(part3.DoubleCountedSide == 'X').sum()}")
print(f"Double Counted food: {(part3.DoubleCountedFood == 'X').sum()}")
print(f"Empty Frames: {(part3.EmptyFrame == 'X').sum()}")
print(f"Total frames: {len(part3)}")
print(f"Total combined frames: {len(part3)+len(part2)+len(part1)}")
part3.head()
```

In [ ]:
```
parts = [part1,part2,part3]
dirty = pd.concat(parts)
len(dirty)
```

Create and save clean dataframes

In [ ]:
```
clean = dirty.drop(dirty[(dirty['DoubleCountedFood'] == 'X') | (dirty['DoubleCountedSide'] == 'X') |
                         (dirty['EmptyFrame'] == 'X')].index)
len(clean)
clean.head()
```

In [ ]:
```
Clean = pd.DataFrame({'TrialID':  [TrialID], 'RawTotalFrames': [part1.iloc[0]['RawTotalFrames']
                    + part2.iloc[0]['RawTotalFrames'] + part3.iloc[0]['RawTotalFrames']],
                     'CleanTotalFrames': [part1.iloc[0]['RawTotalFrames']  + part2.iloc[0]['RawTotalFrames']
                    + part3.iloc[0]['RawTotalFrames']]})
#Clean = pd.DataFrame({'TrialID':  [TrialID], 'RawTotalFrames': [len(dirty)], 'CleanTotalFrames': [len(clean)],
Clean['BMAA_food'] = clean.BMAA_food.sum() + part2.iloc[0]['BMAA_food'] + part3.iloc[0]['BMAA_food']
Clean['Control_food'] = clean.Control_food.sum() + part2.iloc[0]['Control_food'] + part3.iloc[0]['Control_food'
Clean['BMAA_side'] = clean.BMAA_side.sum() +  part2.iloc[0]['BMAA_side'] + part3.iloc[0]['BMAA_side']
Clean['Control_side'] = clean.Control_side.sum() + part2.iloc[0]['Control_side'] + part3.iloc[0]['Control_side'
Clean['FirstBMAA'] = firstBMAA
Clean['FirstControl'] = firstControl
Clean.head()
```

In [ ]:
```
Clean.to_csv(f'{wd}/CleanLocations.csv', mode='a', index=False, header=False)# appends 'cleaned' data to a mast
```

In [ ]:
```
!jupyter nbconvert --to pdf Notebook_name_here.ipynb --output="desired_name_here"
```

In [ ]:
