# Supplementary material for: Experimental evidence challenges the presumed defensive function of a “slow toxin” in cycads
Source: Sci Rep. 2022 Apr 9;12:6013. doi: 10.1038/s41598-022-09298-3 (PMC8994766; doi:10.1038/s41598-022-09298-3)
Supplement: Supplementary file 2 — Supplementary Information 2. [file 41598_2022_9298_MOESM2_ESM.pdf]

Supplemental Materials

Supplementary Table 1

	β	exp(β)	se(β)	95% CI	z	p
Dosage	0.0001790	1.0001790	0.0013080	0.9976-1.003	0.137	0.891
Sex.Male	0.2807925	1.3241788	0.2211452	0.8584-2.043	1.270	0.204
Dosage*Sex.Male	0.0006868	1.0006870	0.0017770	0.9972-1.004	0.386	0.699

Table 1. Output from Cox proportional hazards test, formula = *pupation ~ dosage + sex + dosage*sex)*, n = 132, Sex.Female as reference.

Higher Dosage Bioassay

Thirty larvae were reared as described in the main experiment on one of two diet treatments: artificial diet spiked with 2000 µg/g BMAA or artificial diet without BMAA (control). Despite the higher dosage, we observed no differences in larval survival ($X^2(1, N = 65) = 0$, p = 0.94), development time ($X^2(1, N = 61) = 0.70$, p = 0.41), or pupal weight (t(57) = 0.31, p = 0.76) between treatments (Fig 1). Pupal sex was not recorded. For logistical reasons, preference assays were conducted with fewer larvae (16 trials total) recorded for 8 hours (10:00-18:00) in smaller behavioral arenas. Larvae behaved quite differently than those in the main experiment: rather than exploring the dishes and sampling both diets, 14/16 larvae spent the entire trial period in contact with whichever cube they encountered first. Only one larva encountered both diets, and one larva did not encounter either diet and was therefore excluded from the analysis. Data were analyzed as discrete proportions in which 9/15 larvae fed on control diet and 6/15 larvae fed on BMAA-spiked diet, shifting the mean preference slightly towards control diet (Fig 2). However, this slight preference was not significant (p = 0.61), and given the small sample sizes should be interpreted with caution.

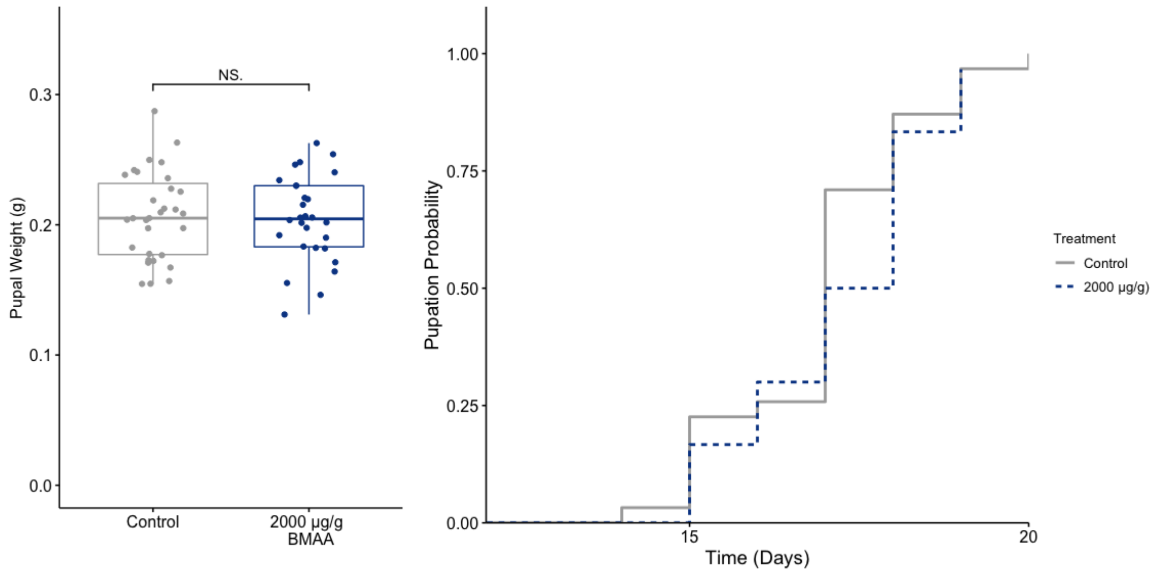

Figure 1. Feeding on BMAA-spiked diet did not significantly affect pupal weight or time to pupation.

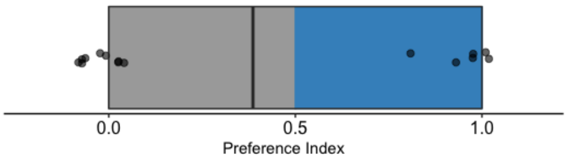

Figure 2. Larvae did not exhibit a significant preference for artificial diet with or without BMAA.
